# Study on the Synergistic Ultrasonic Extraction of Active Components from Loquat Leaves Using Surfactants and Their Mechanism of Action in Weight Loss and Fat Reduction

**DOI:** 10.3390/foods15152727

**Published:** 2026-08-03

**Authors:** Qiang Li, Lulu Jiang, Pinfeng Zhang, Aoyong Tan, Tingting Zhao, Jiale Niu, Weiwei Wang, Xianglong Zhang, Wanli Zhang, Chenxiang Sun

**Affiliations:** Food Laboratory of Zhongyuan, Luohe 462300, China

**Keywords:** loquat leaf extract, obesity, ANN-GA, α-glucosidase inhibition, pancreatic lipase inhibition

## Abstract

The global rise in obesity and associated metabolic disorders has fueled the demand for safe, multi-target therapeutics derived from natural sources. In this study, an artificial neural network combined with a genetic algorithm (ANN-GA), was employed to optimize the ultrasound-assisted extraction of loquat leaf extract (LLE). The optimal parameters—0.6% Tween 80, 328 W ultrasonic power, and 49 °C—enhanced extraction efficiency while minimizing energy consumption and surfactant use. In vitro, LLE exhibited potent dose-dependent inhibition of α-glucosidase (IC_50_ = 3.75 μg/mL, 21.6-fold stronger than acarbose) and pancreatic lipase (IC_50_ = 78.3 μg/mL), indicating strong inhibitory activity against these digestive enzymes. In high-fat diet-induced obese mice, LLE supplementation dose-dependently reduced body weight gain, serum total cholesterol, triglycerides, low-density lipoprotein cholesterol (LDL-C), and fasting blood glucose while elevating high-density lipoprotein cholesterol (HDL-C). In addition, LLE ameliorated liver injury (decreased ALT/AST) and oxidative stress (lowered MDA, increased SOD). These findings highlight LLE as a promising multi-target natural product for managing obesity and related metabolic disorders.

## 1. Introduction

Obesity and its associated metabolic disorders—including hyperlipidemia, type 2 diabetes, and non-alcoholic fatty liver disease (NAFLD)—have emerged as major global public health challenges [1]. Their onset and progression are closely linked to chronic energy surplus, disordered lipid metabolism, and persistent low-grade inflammation [2]. Although existing synthetic weight-loss and lipid-lowering drugs demonstrate some clinical efficacy, they frequently raise safety concerns, carry risks of long-term dependency, and produce adverse effects [3]. Consequently, natural bioactive compounds with proven safety, mild action, and multi-target regulatory properties have increasingly become a research focus in food science and nutritional intervention [4]. Loquat leaves (*Eriobotrya japonica* Thunb.) are a traditional Chinese medicinal and edible plant resource, historically used to clear lung heat, relieve cough, detoxify, and regulate metabolism [5,6]. Modern research indicates loquat leaves are rich in bioactive components, including triterpenoids and polyphenols (such as flavonoids and phenolic acids) [7]. Among these, triterpenoids like ursolic acid and oleanolic acid exhibit significant lipid-regulating, anti-inflammatory, and insulin-sensitizing effects, while polyphenols—particularly flavonoids—show promising antioxidant activity, inhibit lipid absorption, and modulate the gut microbiota [8,9]. However, owing to the hydrophobic nature or high structural stability of these active components, traditional water extraction or organic solvent reflux methods often suffer from low extraction efficiency, high energy consumption, and significant loss of activity, limiting the high-value utilization of loquat leaf resources [10]. Ultrasound-assisted extraction (UAE) disrupts plant cell structures through cavitation effects and enhances solute mass transfer, making it a widely adopted green technology for natural product extraction [11]. Nevertheless, UAE alone remains constrained by solubility limitations and interfacial mass transfer resistance when targeting hydrophobic or tightly bound active compounds. Surfactants—functional additives that markedly reduce interfacial tension and promote the dissolution and migration of hydrophobic substances—have recently been incorporated into plant extraction systems [12]. Food-grade non-ionic surfactants (e.g., Tween and Span series) offer the advantages of high safety and broad applicability [13]. By forming micelles that encapsulate hydrophobic molecules, they can substantially improve the dissolution behavior of target components [14]. The synergistic application of surfactants with ultrasonic technology is expected to enhance both the extraction efficiency and selectivity of loquat leaf bioactive components through the combined effects of physical field intensification and chemical adjuvant action. However, existing studies have largely focused on optimizing single extraction conditions or specific component classes, with limited systematic evaluation of the biological functions of the resulting extracts and their underlying mechanisms.

Therefore, this study uses loquat leaves as the research material to establish a novel synergistic surfactant-enhanced ultrasonic extraction process. A genetic algorithm-backpropagation neural network (GA-BPNN) was introduced for intelligent process optimization. Multiple factors—including surfactant concentration, ultrasonic power, and extraction temperature—were used as inputs, with total phenolic content, total flavonoid content, and ursolic acid content as outputs. Furthermore, using a high-fat diet animal model, the weight-loss and lipid-lowering effects of loquat leaf extract were validated at the systemic level. This study aims to provide a systematic theoretical foundation and technical support for the efficient preparation of loquat leaf bioactive components and their application in functional foods and health interventions.

## 2. Material and Method

### 2.1. Materials

Freshly harvested loquat leaves were purchased from a local supermarket (Luohe City, China). The nonionic surfactant used for extraction was Tween 80 (Polysorbate 80, polyoxyethylene (20) sorbitan monooleate, CAS No. 9005-65-6, E433/INS 433), a food-grade emulsifier obtained from Sigma-Aldrich, (St. Louis, MO, USA). Rutin standard, Trolox standard, dithiothreitol, ABTS, anthrone standard, DPPH, and phloretin were purchased from Shanghai Yuanye Biotechnology Co., Ltd. (Shanghai, China). Ferric chloride, potassium chloride, potassium persulfate, and sodium chloride were purchased from Sinopharm Chemical Reagent Co., Ltd. (Beijing, China). Gallic acid standard (purity ≥ 98%) and ursolic acid standard (purity ≥ 98%) were purchased from Shanghai Titan Technology Co., Ltd. (Shanghai, China). All reagents were of analytical grade.

### 2.2. Preparation of Loquat Leaf Powder

Fresh loquat leaves were thoroughly rinsed and frozen at −80 °C in an ultra-low-temperature freezer. They were then placed in a freezing dryer until the mass remained constant. The freeze-dried loquat leaves were pulverized using a grinder (2000 rpm) and sieved through a 60-mesh screen for later use.

### 2.3. ANN-GA Optimization

**Figure 1:**
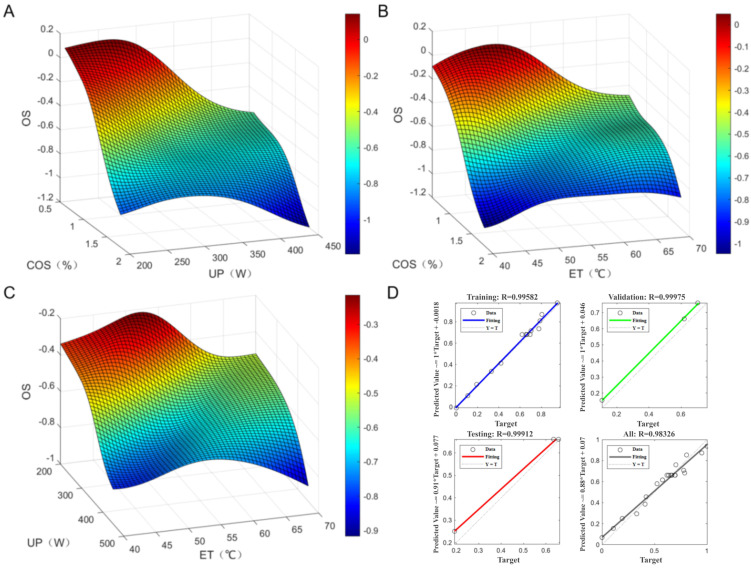
Three-dimensional plot of the relationship between input parameters (Tween 80 concentration, ultrasound power, extraction temperature) and output (OS) parameters in the ANN model (**A**–**C**), and the results of the ANN model (**D**).

The method of Patra et al. [15] was followed with modifications. Comprehensive scores for the 20 sample groups designed by central composite design (CCD) were calculated using the specified formula. One-way ANOVA was performed with Tween 80 concentration, extraction temperature, and ultrasound power as independent variables and the overall score (OS) as the dependent variable. The experimental results were modeled through ANN analysis, as shown in Figure 1. The ANN model comprised an input layer of 3 neurons (Tween 80 concentration, ultrasound power, and extraction temperature), a hidden layer of 10 neurons, and an output layer of 1 neuron pair (OS of samples). The experimental data were randomly divided into three subsets: training (70%), validation (15%), and testing (15%). The Levenberg–Marquardt algorithm was used to iteratively refine the neural network’s weight vectors and biases. Model performance was evaluated using the mean squared error (MSE) and the regression correlation coefficient. Three-dimensional surface plots were generated using MATLAB R2024a software (version number: 24.1.0.2537033). The model was further integrated with genetic algorithms (GA) to optimize the extraction process for loquat leaf extracts, aiming to maximize the output value; the highest (peak) point on the three-dimensional response surface corresponds to the process yielding the optimal comprehensive score.

To comprehensively evaluate the quality of Eriobotrya japonica leaf extract, the entropy weight method was employed to integrate the contents of total flavonoids (*Y*_1_), total phenolics (*Y*_2_), and ursolic acid (*Y*_3_) into a single comprehensive score.

First, positive normalization was performed for each indicator:

$$Y_{ij}^{*}=\frac{Y_{ij}-minY_{j}}{maxY_{j}-minY_{j}}$$

The information entropy of the j-th indicator was calculated as:

$$e_{i}=-\frac{1}{ln\eta }\sum_{i=1}^{\eta }P_{ij}lnP_{iJ},\;P_{ij}=\frac{Y_{ij}^{*}}{\sum_{i=1}^{\eta }Y_{ij}^{*}}$$

The coefficient of variation was dj = 1 – ejdj = 1 − ej, and the weight of each indicator was determined by:

$$W_{j}=\frac{d_{j}}{\sum_{j=1}^{3}d_{j}}$$

Finally, the comprehensive score was obtained as:

$$S_{i}=\sum_{j=1}^{3}W_{j}\times Y_{ij}^{*}$$

$S_{i}$ ranges from 0 to 1, with a higher value indicating better overall quality of the extract. This score served as the unified objective function for subsequent optimization and evaluation.

Furthermore, to balance extraction efficiency with process economics and sustainability, we defined the following eco-efficiency fitness function (*F*) for the GA optimization.

$$F=S_{i}-\alpha \times \frac{C_{surf}}{C_{max}}-\beta \times \frac{P}{P_{max}}$$

where the *C*_surf_ (%) and *P* (W) are the surfactant concentration and ultrasonic power, respectively; *C*_max_ = 1.8% and *P*_max_ = 450 W are the maximum tested levels used for normalization; *α* and *β* are penalty coefficients representing the marginal costs of surfactant usage and electrical energy consumption relative to yield gains. Based on preliminary cost–benefit analysis (raw material prices and local electricity tariffs), we empirically set *α* = 0.15 and *β* = 0.10.

### 2.4. Determination of Total Flavonoids

Total flavonoids were determined using the NaNO_2_-Al(NO_3_)_3_ method [16]. Briefly, 400 μL of the sample solution (raw micellar extract) was transferred to a volumetric flask and diluted to 2.0 mL with deionized water. From this diluted solution, 200 μL was pipetted into a test tube, followed by the sequential addition of 600 μL of 5% NaNO_2_ solution. The mixture was vortexed and allowed to stand for 5 min. Next, 600 μL of Al(NO_3_)_3_ solution was added, and the mixture was allowed to stand for 6 min. Then, 1.8 mL of 4% NaOH solution was added, and the mixture was allowed to stand for 15 min. Absorbance was measured at 510 nm. Rutin was used as the standard (0–2.0 mg/mL) to construct a calibration curve. It should be noted that this colorimetric method primarily detects flavonoids with ortho-dihydroxyl groups and may cross-react with certain phenolic acids; thus, the values represent apparent flavonoid content.

### 2.5. Determination of Total Phenols

Total phenols were determined using the Folin-Ciocalteu method [17]. A 0.1 mL aliquot of the test solution was transferred to a 25 mL stoppered test tube and dilute to 1 mL with water. Then, 1.5 mL of 20% Na_2_CO_3_ solution and 0.5 mL of Folin–Ciocalteu phenol reagent were added. The volume was adjusted to 15 mL, shaken thoroughly, and incubated for 1 h. Absorbance was measured at 760 nm. A calibration curve was constructed using gallic acid as the standard. The Folin–Ciocalteu assay is susceptible to interference from non-phenolic reducing substances (e.g., ascorbic acid, reducing sugars); therefore, the results are expressed as apparent total phenolic content.

### 2.6. Determination of Ursolic Acid

Ursolic acid was quantified by high-performance liquid chromatography (HPLC) [18]. The extract was sonicated with 75% ethanol, centrifuged at 8000 r/min, and the supernatant was collected and filtered through a 0.45 μm microporous membrane. A ZORBAX SB-C18 reversed-phase column (250 mm × 4.6 mm, 5 μm) was used. Gradient elution was performed with acetonitrile and 0.1% phosphoric acid aqueous solution as the mobile phase at a flow rate of 1.0 mL/min. The column temperature was maintained at 30 °C, and the detection wavelength was set to 210 nm. Quantitative analysis was conducted using the external standard method with ursolic acid standard reference material.

### 2.7. In Vitro Enzyme Inhibition Assay of Active Components in Loquat Leaves

#### 2.7.1. α-Glucosidase Inhibitory Activity Assay

α-Glucosidase solution (1.5 U/mL) was prepared in 0.1 mol/L phosphate-buffered saline (PBS, pH 6.8) for enzyme inhibition assays [19]. The extracted LLE powder was dissolved in the same buffer to prepare test solutions at concentrations of 1, 2, 3, 4, and 5 mg/mL. In a 96-well plate, 10 μL of each test solution, 50 μL of PBS, and 20 μL of the 1.5 U/mL enzyme solution were added. The solution was mixed thoroughly and incubated at 37 °C for 10 min. Then, 20 μL of 10 mmol/L PNPG solution was added, shaken well, and incubated at 37 °C for 10 min. The reaction was stopped by adding 30 μL of Na_2_CO_3_ stop solution. The absorbance at 405 nm was immediately measured using a microplate reader. Acarbose solutions were used at the same concentration gradient as the positive control group. The α-glucosidase inhibition rate (AGH) was calculated as follows:

$$AGH\left(\%\right)=\frac{Abs0-Abs1}{Abs0}\times 100$$

where *Abs0* represents the absorbance of the negative control group, and Abs1 represents the absorbance of the test group.

The half-maximal inhibitory concentration (IC_50_) is defined as the sample concentration at which α-glucosidase activity is inhibited by 50%.

#### 2.7.2. Assay for Inhibition of Pancreatic Lipase Activity

p-Nitrophenyl palmitate was dissolved in DMSO to obtain a 50 mmol/L solution. The lyophilized extract powder was dissolved in deionized water to prepare a 5 mg/mL test solution. Pancreatic lipase was dissolved in Tris buffer (pH = 7.4) to a concentration of 10 mg/mL and centrifuge at 4 °C and 13,000 rpm for 8 min, and the supernatant was collected. The total reaction volume was 1.84 mL, comprising 0.2 mL test solution, 0.2 mL enzyme solution, 1.4 mL Tris buffer (pH = 7.4), and 0.4 mL substrate solution. The mixture was incubated at 37 °C for 20 min, and absorbance was measured at 405 nm, using deionized water as the blank control. The pancreatic lipase inhibition rate was calculated using the following formula:

$$PPLIR\left(pancreatic\;lipase\;inhibition\;rate,\%\right)\;=\frac{Abs0-(Abs1-Abs2)}{Abs0}\times 100$$

where *Abs*0 represents the absorbance value without adding the sample; *Abs*1 represents the absorbance value after adding the inhibitor; and *Abs*2 represents the absorbance value after adding the inhibitor but without adding the substrate and enzyme solution.

### 2.8. Establishment and Testing of Animal Models

#### 2.8.1. Establishment of a High-Fat-Diet Obesity Mouse Model and Drug Administration

Sixty 4-week-old male C57BL/6J mice were selected and housed in plastic cages (four mice per cage) under controlled conditions (temperature: 25 ± 2 °C; humidity: 55% ± 10%; 12 h light cycle: 12 h dark cycle). After 7 days of acclimation, mice were randomly divided into five groups (*n* = 12 per group, total 60 mice): the negative control group received a low-fat diet (NC, 10% energy from fat); the experimental control group received a high-fat diet (HFD, 60% calories from fat); the positive control group received a high-fat diet plus orlistat 60 mg/kg via gavage (OL); and two treatment groups received a high-fat diet supplemented with either low-dose (LLE-L) or high-dose (LLE-H) loquat leaf extract, with doses determined based on prior in vitro enzyme activity inhibition assays. The sample size (*n* = 12 per group) was determined by an a priori power analysis using G*Power 3.1 software (effect size f = 0.40, α = 0.05, power = 0.80), which estimated a minimum of 10 mice per group for the primary outcome variables (body weight and serum lipid levels). Considering a potential 15–20% attrition rate during the 12-week experimental period, we enrolled 12 mice per group to ensure adequate statistical power. The entire experimental period lasted 12 weeks, during which basic physiological indicators such as body weight changes and food intake were regularly monitored and recorded. At the end of the experiment, mice were fasted for 12 h (with free access to water) and then euthanized under anesthesia. Blood samples were collected, and liver tissue was immediately excised on ice. A portion of fresh liver tissue was stored at −80 °C for Western blot analysis, while the remainder was fixed in 4% paraformaldehyde for histological analysis.

#### 2.8.2. Biochemical Parameter Measurement

Blood glucose levels were monitored using a portable blood glucose meter (Bayer HealthCare LLC, Mishawaka, IN, USA). Levels of TC, TG, HDL-C, LDL-C, MDA, SOD, ALT, and AST in serum and cell extracts were detected using commercial kits [20]. All measurements were performed on a SpectraMax Plus Biochemical Analyzer (Molecular Devices, Sunnyvale, CA, USA) according to the manufacturer’s instructions.

#### 2.8.3. Inflammatory Cytokine Level Assay

Inflammatory cytokine levels, including IL-1β, IL-2, IL-6, and TNF-α, were measured and calculated using a mouse enzyme-linked immunosorbent assay (ELISA) kit.

#### 2.8.4. Serum Insulin Level Measurement

Serum insulin levels were measured using an ELISA kit (Wuhan Jiyinmei Technology Co., Ltd., Wuhan, China) [20]. The formula for calculating the HOMA-IR index, an indicator of insulin resistance in the steady-state model, was as follows:

$$HOMA-IR\;indexe\;=\frac{A_{1}\times A_{2}}{22.51}$$

where *A*1 and *A*2 represent fasting blood glucose and fasting insulin levels, respectively.

### 2.9. Statistical Analysis

All data are expressed as mean ± SEM and analyzed using SPSS software (version 20.0) and Origin 2025. One-way analysis of variance (ANOVA) was performed, with *p* < 0.05 as the significance level (Duncan’s multiple range test).

## 3. Results and Discussion

### 3.1. Optimization of the Extraction Process Based on ANN-GA

We conducted 20 experiments using CCD designs (Table 1) to develop an ANN model for predicting LLE quality. The results indicated that the composite score exhibits a continuous upward trend with increasing surfactant concentration (COS) (Table 1). When the concentration increased from 0.6% to 1.6%, the *S* value rose from 0.000 to 0.950–1.000. This may be attributed to surfactants disrupting the cell wall-vacuole interface through solubilization, enhancing membrane lipid fluidity, and facilitating the dissolution of intracellular triterpenic acids, flavonoids, and phenolic compounds [21]. Concurrently, the hydrophobic tails of surfactant molecules form mixed micelles with target components, increasing the effective loading capacity in the extract. However, beyond 1.6% concentration (Experiment 14, A = 1.8%), the S value did not increase further (0.805), suggesting excessively high surfactant concentrations may generate excessive foam, complicate subsequent separation, and potentially form complexes with phenolic acids under acidic conditions, leading to a slowdown in yield improvement. Ultrasound power (UP) exhibited a significant positive effect on the comprehensive score, but excessively high power slightly decreased the OS value. This indicates that cavitation effects at appropriate power levels can instantaneously generate high-temperature, high-pressure microjets, efficiently disrupting the trichomes and thick-walled cells on loquat leaves, shortening mass transfer distances, and promoting surfactant diffusion toward the solid-liquid interface [22]. However, excessively high power (>400 W) may cause localized overheating, inducing thermal isomerization or degradation of phenolic compounds and triterpenic acids. Moreover, excessive cavitation tends to increase dissolved oxygen in the system, accelerating the oxidative polymerization of certain polyphenols. The effect of extraction temperature (ET) on the comprehensive score follows a quadratic curve, first increasing and then decreasing. Within the 40–65 °C range, the OS value increases with rising temperature, peaking at 65 °C. However, when the temperature rises to 70 °C (Experiment 16), the S value drops back to 0.524. This may be attributed to the dual effects of elevated temperature: on one hand, it enhances molecular thermal motion and lowers the critical micelle concentration (CMC), thereby facilitating micelle formation; concurrently, elevated temperature increases micellar aggregation numbers and core hydrophobicity, improving solubilization capacity; while on the other hand, pentacyclic triterpenoids like ursolic acid undergo decarboxylation or oxidation at elevated temperatures. Additionally, thermally unstable chlorogenic acid and caffeic acid derivatives within total phenols degrade significantly faster at 70 °C. Furthermore, excessively high temperatures accelerate pectin dissolution from loquat leaf residues, increasing extract viscosity and hindering solid-liquid separation. From the interaction analysis, the combination of high surfactant concentration (≥1.6%) and high ultrasonic power (≥400 W) demonstrated a pronounced synergistic effect (Experiments 9, 20), with S values exceeding 0.95. Conversely, at low concentrations (0.8%), even with increased power or optimized temperature, S values remained below 0.58 (Experiments 8, 12, 17). This indicates that surfactant concentration is the critical threshold for breaching the cell barrier, while ultrasonic power further enhances mass transfer upon effective solubilization. The coefficient of variation (CV) for the S value in the 6 central point experiments (COS = 1.2%, UP = 325 W, ET = 55 °C) was 5.1%, demonstrating good reproducibility of this system.

Furthermore, the GA-ANN optimization, using the eco-efficiency fitness function (F), identified the optimal parameters at a surfactant concentration of 0.6%, ultrasonic power of 328 W, and extraction temperature of 49 °C. Although this condition corresponds to a slightly lower raw OS (predicted ~0.96) compared to the highest-yield CCD run at 1.6%/400 W/65 °C (experimental OS = 0.985), it provides a markedly superior fitness score (F) due to the ~62.5% reduction in surfactant usage and ~18% reduction in ultrasonic energy consumption. This balance arises because the fitness function penalizes the steep marginal costs of exceeding 0.6% surfactant (Experiments 14–20), where further increases in OS per unit of surfactant become progressively smaller. This may stem from GA-ANN’s ability to fully model nonlinear interactions among factors in continuous solution space. It identified synergistic effects achievable at lower surfactant concentrations: moderately increasing ultrasonic power while maintaining optimal temperature enables efficient cell wall disruption and complete extraction of active components. Where ultrasonic cavitation compensates for the solubilization limitations of surfactants, while the mild 49 °C condition prevents thermal degradation of triterpenic acids and phenolic compounds. Furthermore, although excessively high surfactant concentrations (>1.2%) can further increase yield, they significantly elevate extraction solution viscosity and post-processing difficulty, potentially introducing interfacial adsorption losses. GA-ANN achieves a balance between extraction efficiency and process economics through global optimization, rendering this optimized result more promising for practical application.

### 3.2. Enzyme Activity Inhibition

#### 3.2.1. Inhibition of α-Glucosidase Activity by LLE

**Figure 2:**
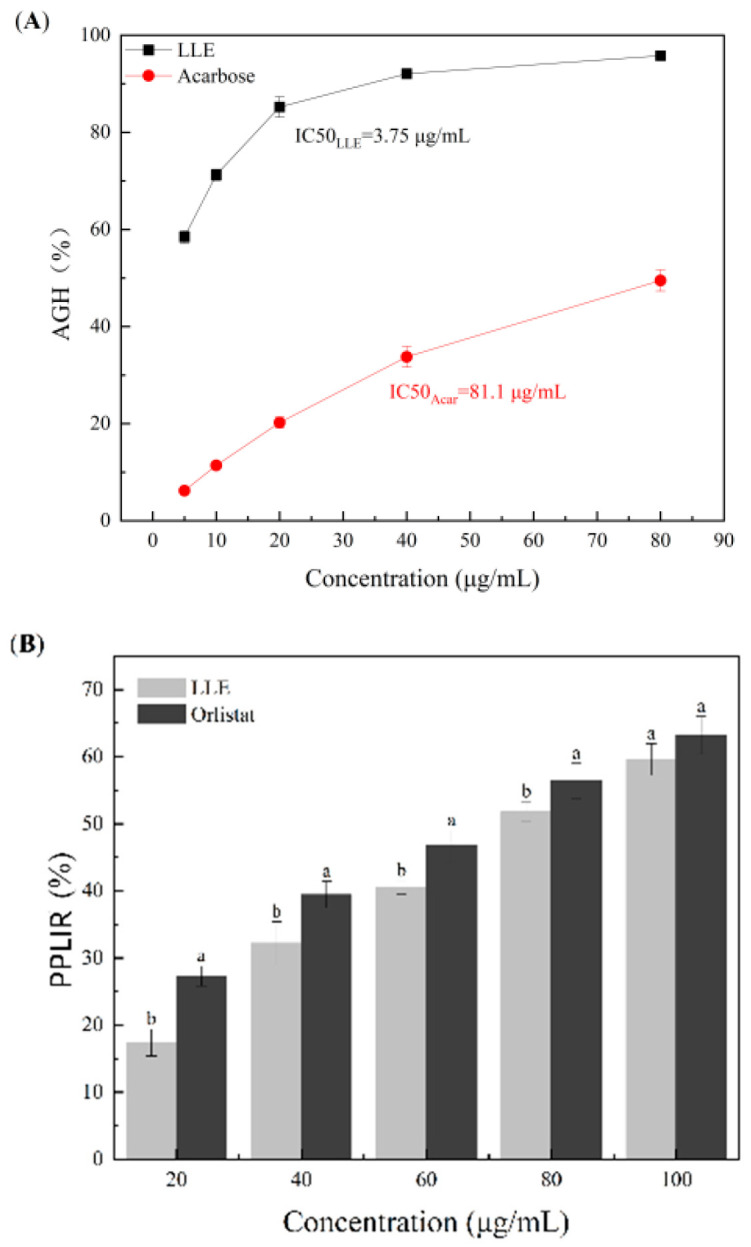
Inhibitory effects of different LLE concentrations on enzymatic activity (**A**): AGH, (**B**): PPLIR. Different lowercase letters denote significant differences between groups at *p* < 0.05; statistically significant differences were observed in the individual comparisons of LLE and orlistat at different concentrations.

The inhibitory effect of LLE on α-glucosidase is shown in Figure 2A. Within the tested concentration range of 5–80 μg/mL, LLE exhibited potent dose-dependent inhibitory activity, with inhibition rates increasing from 58.48% to 95.77%. Nonlinear regression analysis of the dose-response curve using a four-parameter logistic model yielded an IC_50_ value of 3.75 μg/mL (95% CI: 2.98–4.52 μg/mL, R^2^ = 0.991). Notably, because the inhibition rate at the lowest tested concentration (5 μg/mL) already exceeded 50%, this IC_50_ value was obtained by extrapolation from the fitted sigmoidal curve. In contrast, the positive control drug acarbose exhibited inhibition rates increasing only from 6.19% to 49.49% within the same concentration range, with an IC_50_ of 81.1 μg/mL (95% CI: 72.5–90.6 μg/mL, R^2^ = 0.985). From a mechanism perspective, this potent inhibition likely stems from bioactive components in loquat leaves, such as flavonoids and triterpenic acids. These compounds bind to amino acid residues in the active site of α-glucosidase through hydrogen bonds and hydrophobic interactions, competitively blocking the enzyme’s binding site for starch or altering the enzyme’s conformation to inactivate it, thereby effectively delaying starch hydrolysis and glucose release [23].

#### 3.2.2. Inhibition of LLE on Pancreatic Lipase Activity

The in vitro inhibitory effect of LLE on pancreatic lipase activity is shown in Figure 2B. Results indicate that at 20 μg/mL, LLE exhibited a 17.4% inhibition rate against PL activity. Inhibition progressively increased with rising concentrations, reaching 59.6% at 100 μg/mL. The calculated half-maximal inhibitory concentration (IC_50_) was approximately 78.3 μg/mL, demonstrating clear dose-dependent inhibition. The positive control, orlistat, exhibited an inhibition rate ranging from 27.31% to 63.2% within the same concentration range, with an IC_50_ of approximately 66.8 μg/mL. Comparative potency analysis indicates that although LLE exhibits slightly lower PL inhibitory activity than orlistat, it demonstrates strong inhibitory potential. Notably, inhibition rates increase rapidly within the low concentration range (20–60 μg/mL), suggesting the presence of high-affinity active components. Inhibition of PL by plant extracts is typically attributed to their phenolic and flavonoid compounds. Sandhiya et al. studied *P. benghalensis*, confirming that the water-methanol extract obtained via ultrasonic-assisted extraction was rich in phenolic and flavonoid components [24]. At 100 μg/mL, it achieved a PL inhibition rate of 57%, with an IC_50_ of 86.2 μg/mL, consistent with the findings of this study. Phenolic compounds competitively inhibit triglyceride substrate binding and hydrolysis by forming hydrogen bonds between their hydroxyl groups and the serine-histidine-aspartic acid triplet in the lipase active site, while their hydrophobic backbone embeds into the enzyme’s hydrophobic pocket. Flavonoids (e.g., quercetin, kaempferol) and triterpenic acids abundant in LLE have also been reported in multiple studies to exhibit PL inhibitory activity. Their mechanisms of action may involve interactions with the enzyme’s active site and conformational changes [25].

### 3.3. Effects of LLE on Total Cholesterol (TC) and Triglycerides (TG) in Obese Mice

**Figure 3:**
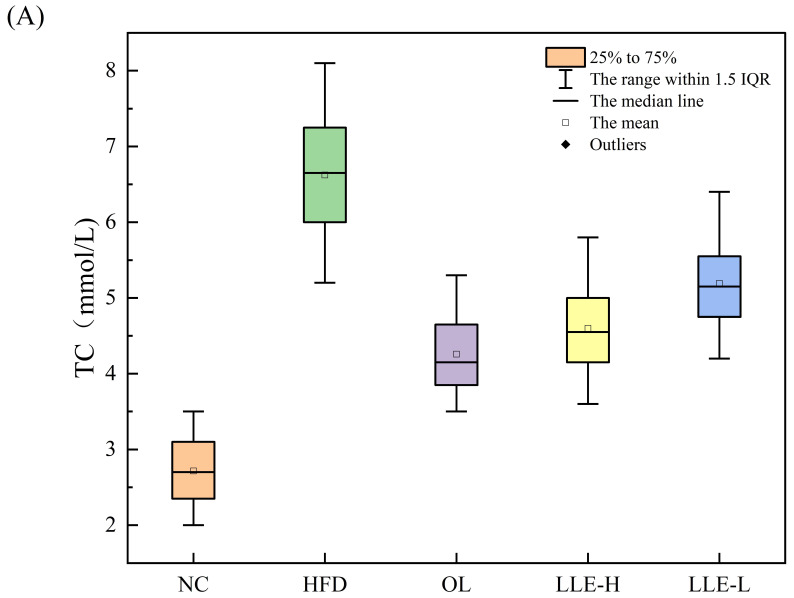

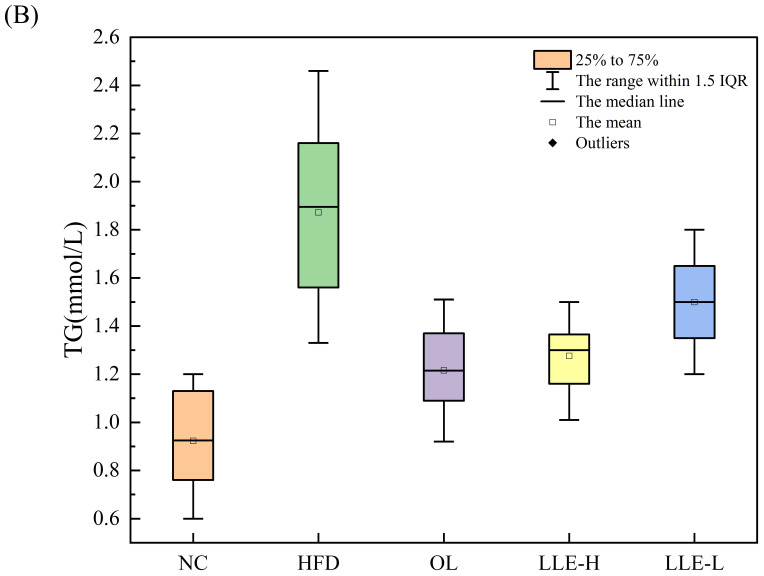
Effects of LLE on total cholesterol (**A**) and triglycerides (**B**) in obese mice.

The effects of LLE on total cholesterol (TC) and triglycerides (TG) in mice are shown in Figure 3. Results indicate that TC and TG levels in the high-fat diet (HFD) group were significantly elevated to 6.90 ± 0.80 mmol/L and 1.90 ± 0.30 mmol/L, respectively, compared to the normal chow (NC) group (TC 2.70 ± 0.40 mmol/L, TG 0.90 ± 0.15 mmol/L). This represents approximately 2.5-fold and 2.1-fold increases, confirming that the high-fat diet successfully induced lipid metabolism disorders. In the orlistat positive control group (OL), TC and TG levels decreased to 4.50 ± 0.50 mmol/L and 1.20 ± 0.15 mmol/L, respectively, with inhibition rates of approximately 35% and 37%. This aligns with orlistat’s pharmacological mechanism as a potent pancreatic lipase inhibitor that reduces intestinal triglyceride digestion and absorption. The high-dose loquat leaf extract group (LLE-H) exhibited TC and TG levels of 4.70 ± 0.60 mmol/L and 1.30 ± 0.15 mmol/L, respectively, showing no statistically significant difference from the OL group, while the low-dose group (LLE-L) showed values of 5.30 ± 0.60 mmol/L and 1.50 ± 0.18 mmol/L, demonstrating a clear dose-dependent effect. The lipid-lowering effect of LLE is primarily attributed to its rich flavonoid (e.g., quercetin, kaempferol) and triterpenic acid (e.g., corosolic acid, ursolic acid) components. These compounds not only directly inhibit pancreatic lipase activity but also downregulate hepatic SREBP-1c and FAS expression by activating the AMPK pathway, thereby reducing endogenous lipid synthesis. Additionally, polyphenolic substances in LLE bind to bile acids, promoting cholesterol excretion and further lowering TC levels [26]. Compared to orlistat, LLE demonstrates comparable efficacy in reducing TG levels, suggesting its potential advantage as a multi-targeted natural product in improving hyperlipidemia.

### 3.4. Effects of LLE on High-Density Lipoprotein Cholesterol and Low-Density Lipoprotein Cholesterol in Obese Mice

**Figure 4:**
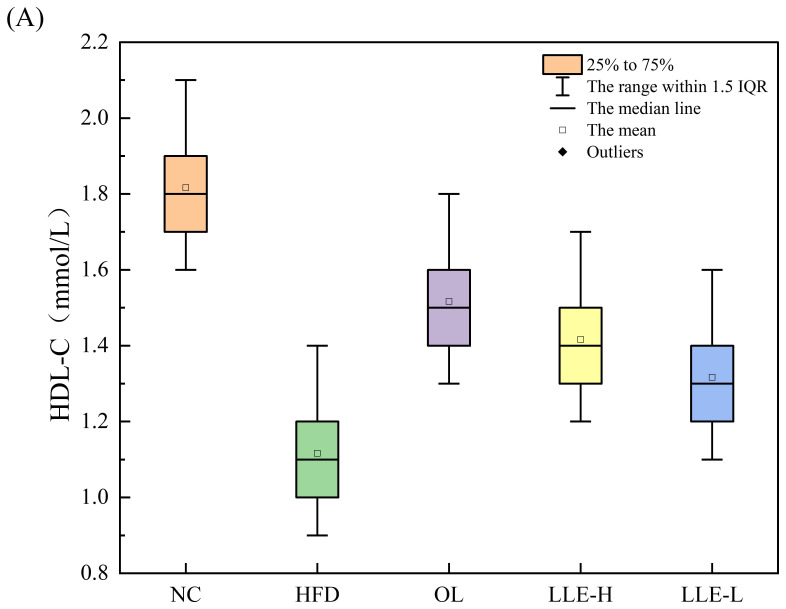

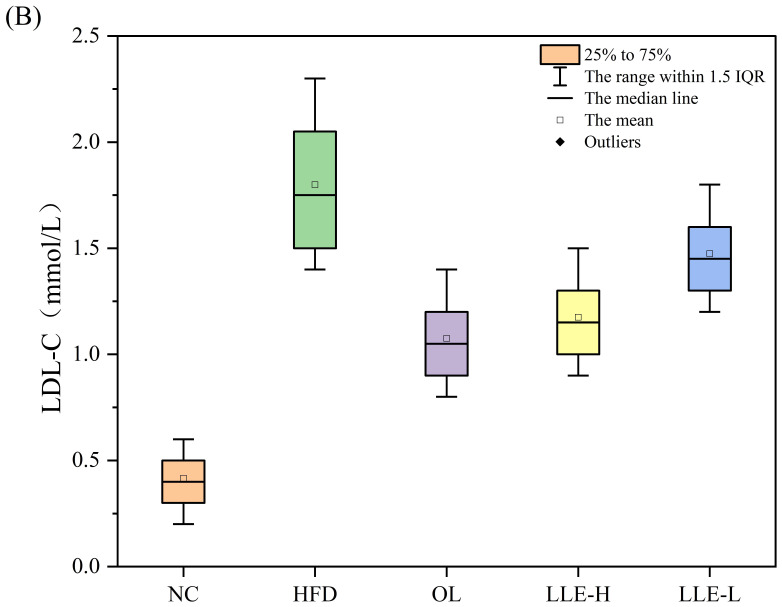
Effects of LLE on high-density lipoprotein cholesterol (**A**) and low-density lipoprotein cholesterol (**B**) in obese mice.

High-density lipoprotein cholesterol (HDL-C) and low-density lipoprotein cholesterol (LDL-C) are key indicators for assessing cardiovascular risk. As shown in Figure 4, the HFD group exhibited a significant decrease in HDL-C to 1.10 ± 0.12 mmol/L, while LDL-C increased to 1.80 ± 0.25 mmol/L. This led to a substantial rise in the atherosclerosis index (LDL-C/HDL-C), indicating that a high-fat diet induces an atherogenic lipoprotein profile. Following orlistat intervention, HDL-C rebounded to 1.50 ± 0.12 mmol/L, and LDL-C decreased to 1.10 ± 0.18 mmol/L yet remained lower than the NC group (HDL-C 1.80 ± 0.15 mmol/L, LDL-C 0.40 ± 0.10 mmol/L). The LLE-H group achieved HDL-C of 1.40 ± 0.12 mmol/L and LDL-C of 1.20 ± 0.18 mmol/L, demonstrating improvement comparable to the OL group. The LLE-L group showed HDL-C of 1.30 ± 0.14 mmol/L and LDL-C of 1.50 ± 0.20 mmol/L. The mechanism by which LLE elevates HDL-C may involve upregulating hepatic ApoA-I expression and increasing lecithin cholesterol acyltransferase (LCAT) activity, thereby promoting cholesterol reverse transport. Concurrently, flavonoids in LLE may accelerate LDL-C clearance by inhibiting PCSK9 expression and increasing LDL receptor density on hepatocyte surfaces. Compared to orlistat’s sole inhibition of fat absorption, LLE demonstrated more pronounced HDL-C-enhancing effects, potentially attributable to its antioxidant and anti-inflammatory properties—as oxidative stress and inflammation suppress HDL-C synthesis and function.

### 3.5. Effects of LLE on Alanine Transaminase and Aspartate Transaminase in Obese Mice

**Figure 5:**
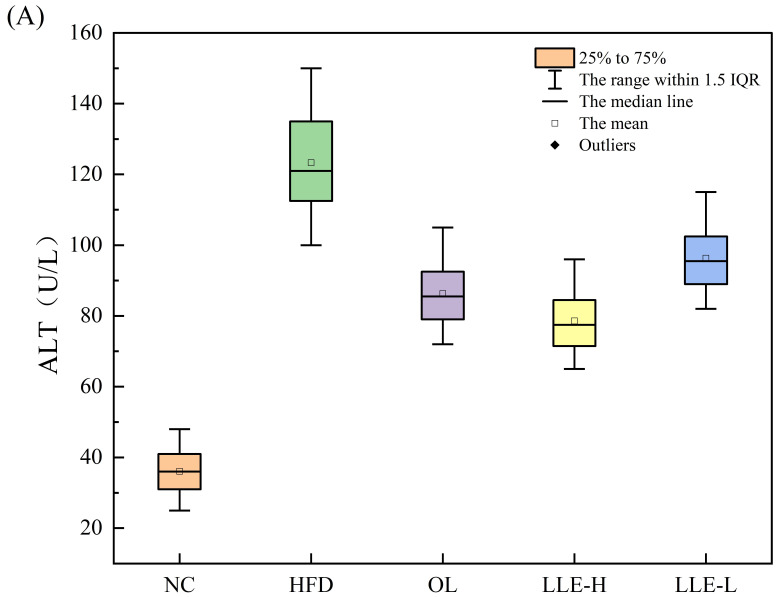

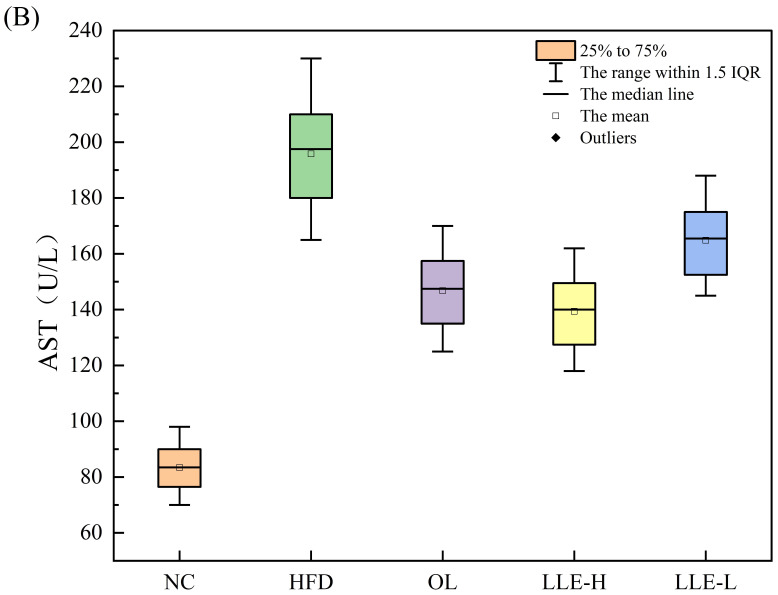
Effects of LLE on ALT (**A**) and AST (**B**) in obese mice.

Alanine transaminase (ALT) and aspartate transaminase (AST) are sensitive indicators reflecting hepatocyte damage. As shown in Figure 5, ALT and AST levels in the HFD group rose to 125 ± 15 U/L and 195 ± 20 U/L, respectively, significantly higher than those in the NC group (ALT 35 ± 6 U/L, AST 82 ± 8 U/L). This indicates that a long-term high-fat diet has caused significant liver damage, consistent with the pathological features of non-alcoholic fatty liver disease (NAFLD). After orlistat intervention, ALT and AST decreased to 88 ± 10 U/L and 148 ± 15 U/L, respectively, but remained above normal levels. The LLE-H group showed ALT and AST levels of 78 ± 9 U/L and 138 ± 12 U/L, respectively, which were lower than the OL group and approached the normal range. The LLE-L group exhibited levels of 95 ± 11 U/L and 165 ± 18 U/L, demonstrating a dose-dependent improvement. The hepatoprotective effects of LLE stem from multiple mechanisms. First, it reduces hepatic lipid load and alleviates hepatic steatosis by inhibiting lipase and decreasing intestinal fat absorption. Second, phenolic compounds in LLE (e.g., eugenol, chlorogenic acid) exhibit potent antioxidant activity, scavenging free radicals, inhibiting lipid peroxidation, and reducing malondialdehyde (MDA) production, thereby preserving hepatocyte membrane integrity. Third, LLE downregulates pro-inflammatory factors (TNF-α, IL-6) expression, inhibits Kupffer cell activation, and alleviates hepatic inflammatory responses [27]. Compared to orlistat, LLE demonstrates superior efficacy in lowering transaminase levels, likely due to its triple synergistic effects of lipid-lowering, antioxidant, and anti-inflammatory actions.

### 3.6. Effects of LLE on Malondialdehyde (MDA) and Superoxide Dismutase (SOD) in Obese Mice

**Figure 6:**
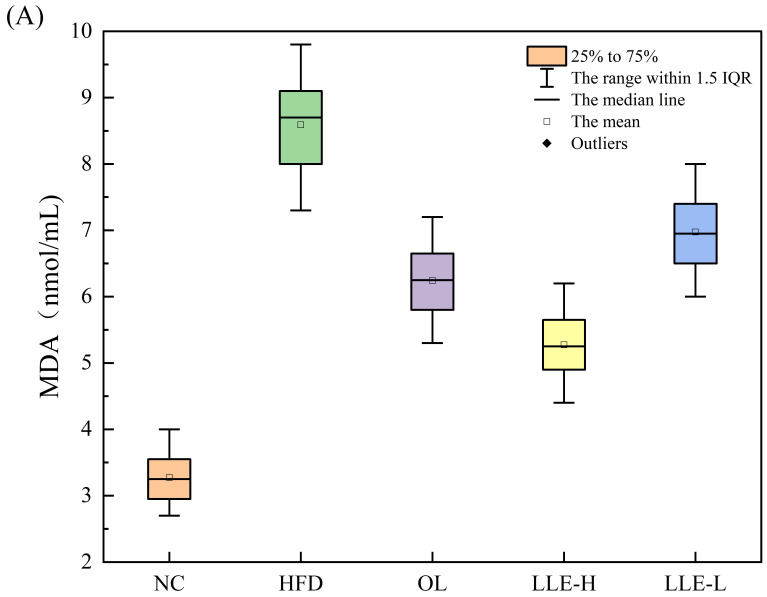

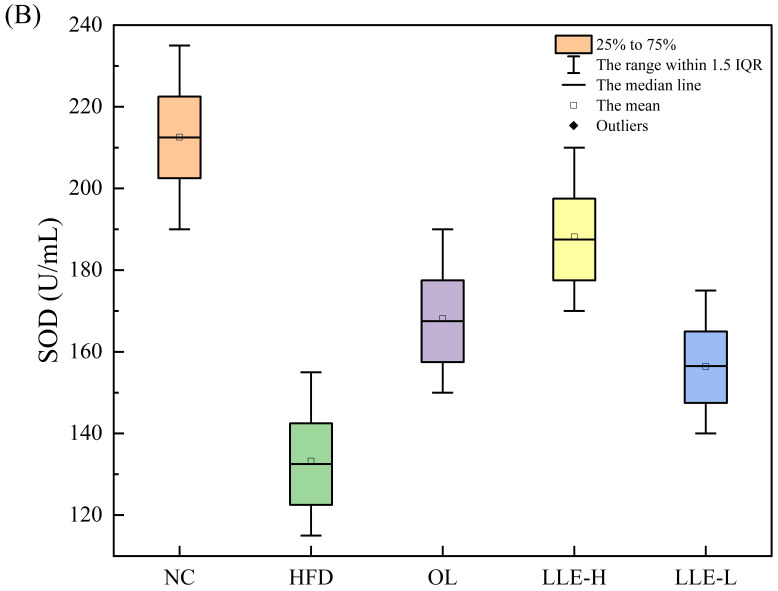
Effects of LLE on MDA (**A**) and SOD (**B**) in obese mice.

Malondialdehyde (MDA) is the end product of lipid peroxidation, reflecting the degree of oxidative stress. Superoxide dismutase (SOD) is the body’s primary antioxidant enzyme, scavenging superoxide anion radicals. As shown in Figure 6, the HFD group exhibited elevated MDA levels at 8.60 ± 0.70 nmol/mL and decreased SOD activity at 132 ± 12 U/mL, indicating an oxidative stress state induced by the high-fat diet. After orlistat intervention, MDA decreased to 6.40 ± 0.60 nmol/mL, and SOD rebounded to 168 ± 14 U/mL, showing limited improvement. In the LLE-H group, MDA significantly decreased to 5.40 ± 0.50 nmol/mL, and SOD increased to 190 ± 15 U/mL, approaching NC group levels (MDA 3.30 ± 0.40 nmol/mL, SOD 215 ± 15 U/mL). The LLE-L group showed MDA at 7.00 ± 0.65 nmol/mL and SOD at 158 ± 13 U/mL. LLE’s potent antioxidant activity stems from its rich polyphenolic constituents. Compounds like quercetin can chelate transition metal ions, directly scavenge free radicals, and activate the nuclear factor E2-related factor 2 (Nrf2) pathway, thereby upregulating antioxidant enzymes such as SOD and glutathione peroxidase (GPx). Triterpenoid compounds like corosolic acid also reduce reactive oxygen species (ROS) production by inhibiting NADPH oxidase activity. Compared to orlistat, LLE demonstrates greater efficacy in lowering MDA levels and enhancing SOD activity, as it not only reduces lipid peroxidation substrates (lipids) but also directly strengthens endogenous antioxidant defense systems [28].

### 3.7. Effects of LLE on Fasting Blood Glucose in Obese Mice

**Figure 7:**
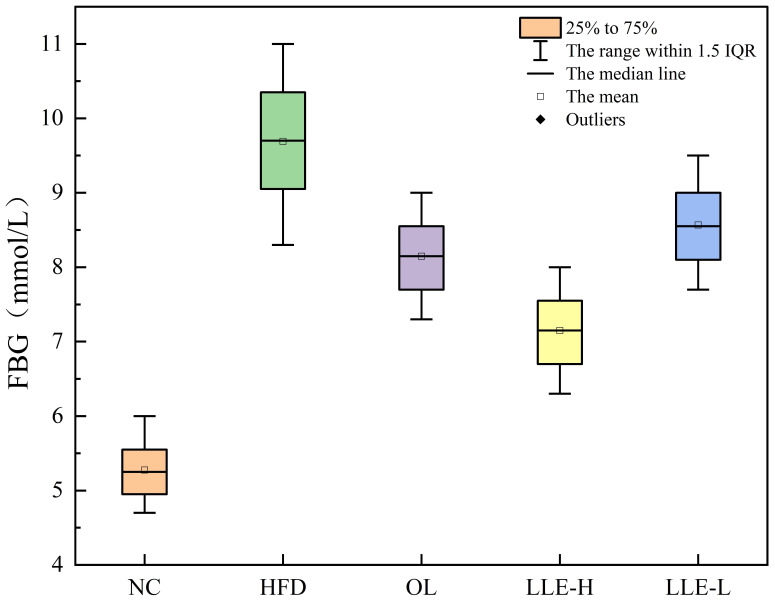
Effects of LLE on FBG in obese mice.

Fasting blood glucose (FBG) reflects glucose homeostasis under basal conditions. As shown in Figure 7, the HFD group exhibited an elevated FBG level of 9.80 ± 0.80 mmol/L, meeting the diagnostic criteria for diabetes, indicating that high-fat diet-induced insulin resistance had disrupted glucose regulation. Following orlistat intervention, FBG decreased to 8.30 ± 0.50 mmol/L, a reduction of approximately 15%, primarily attributed to weight loss and improved lipotoxicity indirectly enhancing insulin sensitivity. The LLE-H group exhibited a significant FBG reduction to 7.30 ± 0.45 mmol/L, a 25% decrease, outperforming orlistat, while the LLE-L group recorded 8.50 ± 0.55 mmol/L. LLE’s hypoglycemic mechanism is more direct. First, its rich flavonoid compounds (e.g., kaempferol) competitively inhibit α-glucosidase and α-glucosidase activity, delaying intestinal carbohydrate digestion and absorption to reduce postprandial blood glucose peaks [29]. Second, triterpenoid components like corosolic acid activate the AMPK pathway, promoting GLUT4 translocation to the cell membrane and enhancing glucose uptake and utilization in peripheral tissues [30]. Third, LLE’s antioxidant effects mitigate oxidative stress damage to pancreatic β-cells, thereby protecting insulin secretion function [31].

### 3.8. Effects of LLE on Inflammatory Factors in Obese Mice

**Figure 8:**
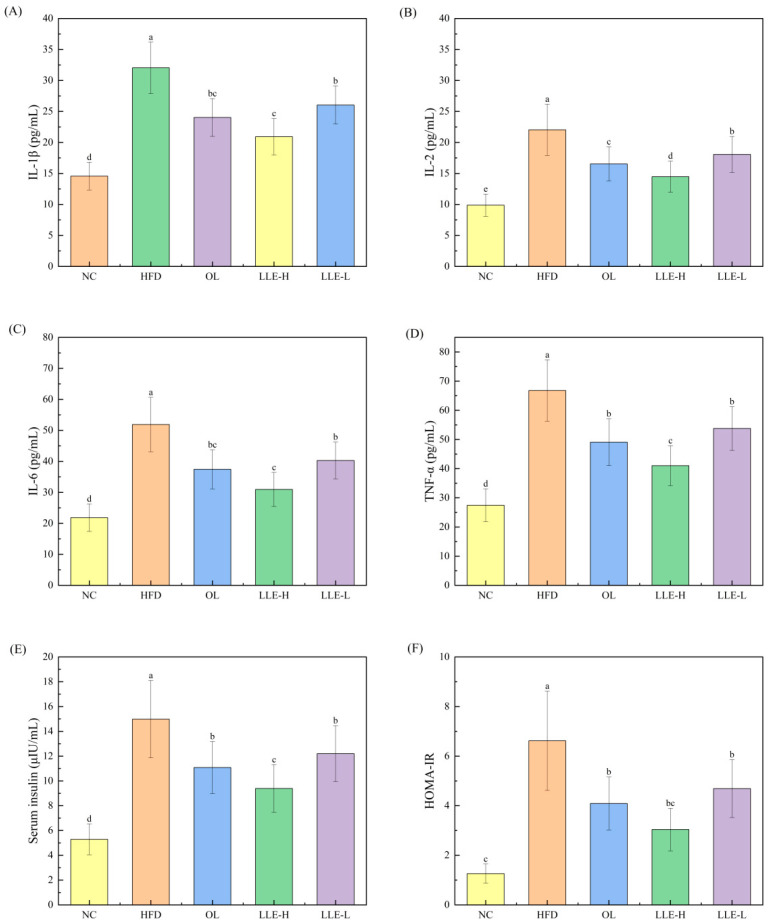
Effects of LLE on inflammatory factors (**A**–**D**), serum insulin levels (**E**), and HOMA-IR (**F**) in obese mice. Different lowercase letters represent significant differences among groups at *p* < 0.05.

Chronic low-grade inflammation is a core pathological features of obesity and its associated metabolic disorders. Macrophage infiltration in adipose tissue and increased pro-inflammatory cytokine secretion directly interfere with insulin signaling and promote the development of non-alcoholic fatty liver disease (NAFLD) [32]. The effects of LLE on levels of inflammatory cytokines interleukin-1β (IL-1β), interleukin-2 (IL-2), interleukin-6 (IL-6), and tumor necrosis factor-α (TNF-α) in high-fat diet-induced obese mice are shown in Figure 8A–D. Results revealed that mice in the high-fat diet (HFD) group exhibited a typical pro-inflammatory state: IL-1β, IL-2, IL-6, and TNF-α levels increased to 32.5 ± 3.5 pg/mL, 22.8 ± 3.2 pg/mL, 52.5 ± 6.5 pg/mL, and 68.5 ± 8.5 pg/mL, respectively, representing increases of 1.2-fold, 1.3-fold, 1.5-fold, and 1.5-fold compared to the normal diet (NC) group (14.8 ± 2.2 pg/mL, 9.8 ± 1.5 pg/mL, 21.2 ± 3.5 pg/mL, 27.5 ± 4.5 pg/mL). This confirmed that the high-fat diet successfully induced a systemic inflammatory response. Following intervention in the orlistat positive control group (OL), the four inflammatory factors decreased to 24.5 ± 2.8 pg/mL, 17.2 ± 2.4 pg/mL, 37.8 ± 4.8 pg/mL, and 48.5 ± 6.5 pg/mL, respectively, representing reductions of approximately 25–29%, suggesting that inhibiting fat absorption, reducing body weight, and alleviating lipid load may mitigate inflammation to some extent. The loquat leaf extract intervention group demonstrated more pronounced anti-inflammatory effects: in the high-dose group (LLE-H), IL-1β, IL-2, IL-6, and TNF-α decreased to 21.2 ± 2.5 pg/mL, 15.2 ± 2.2 pg/mL, 31.2 ± 4.2 pg/mL, and 40.2 ± 5.8 pg/mL, respectively; while the low-dose group (LLE-L) showed levels of 26.5 ± 3.0 pg/mL, 18.5 ± 2.6 pg/mL, 40.2 ± 5.2 pg/mL, and 52.8 ± 7.2 pg/mL, respectively, demonstrating a clear dose-dependent relationship. Notably, all inflammatory marker levels in the LLE-H group were significantly lower than those in the OL group (*p* < 0.05), indicating that its anti-inflammatory effects stem not only from lipid-lowering actions but also from direct modulation of inflammatory pathways. The flavonoids (e.g., quercetin, kaempferol) and triterpenic acids (e.g., corosolic acid, ursolic acid) abundant in LLE can inhibit the production and release of inflammatory mediators through multiple targets. These compounds can downregulate the transcriptional expression of IL-1β, IL-6, and TNF-α by inhibiting the activity of the IκB kinase complex and blocking NF-κB nuclear translocation. Flavonoids inhibit NF-κB and AP-1 signaling pathways downstream of the T-cell receptor, reducing IL-2 gene transcription while indirectly suppressing effector T-cell function by increasing regulatory T-cell (Treg) proportions. Triterpenic acids block TNF-α binding to TNFR1 or downregulate TNFR1 expression, disrupting inflammatory signal amplification at the receptor level. Furthermore, LLE’s potent antioxidant activity scavenges reactive oxygen species (ROS), which serve as crucial second messengers activating multiple inflammatory signaling pathways. This mechanism is directly corroborated by the observed reduction in malondialdehyde (MDA) and elevation of superoxide dismutase (SOD) in this study.

### 3.9. Effects of LLE on Serum Insulin Levels and HOMA-IR in Obese Mice

Insulin resistance is a pivotal step in the progression from obesity to type 2 diabetes, characterized by reduced insulin sensitivity in peripheral tissues and compensatory increased secretion by pancreatic β-cells, ultimately leading to hyperinsulinemia and impaired glucose homeostasis [33]. The effects of LLE on insulin sensitivity in high-fat diet (HFD)-induced obese mice are shown in Figure 8E,F. Results indicate that fasting serum insulin levels in the HFD group rose to 15.2 ± 2.5 μIU/mL, significantly higher than the NC group (5.2 ± 0.8 μIU/mL) (*p* < 0.01), with an HOMA-IR index of 6.6 ± 1.2—approximately 5.5 times that of the NC group (1.2 ± 0.2) (*p* < 0.001)—indicating successful establishment of significant insulin resistance and hyperinsulinemia in the model. Following orlistat intervention, insulin levels decreased to 11.2 ± 1.8 μIU/mL, and HOMA-IR decreased to 4.3 ± 0.8, representing reductions of 26% and 35%, respectively, suggesting partial improvement in insulin sensitivity through reduced lipid load. The LLE intervention group demonstrated more pronounced improvements: the high-dose group (LLE-H) showed insulin levels reduced to 9.5 ± 1.6 μIU/mL and HOMA-IR decreased to 3.2 ± 0.6, representing reductions of 37% and 52%, respectively; while the low-dose group (LLE-L) recorded 12.5 ± 2.0 μIU/mL and 4.8 ± 1.0, respectively, demonstrating clear dose dependency. Both metrics in the LLE-H group significantly outperformed the OL group (*p* < 0.05), indicating superior efficacy in improving insulin resistance. The flavonoids abundant in LLE competitively inhibit α-glucosidase and α-glucosidase activity, delaying intestinal carbohydrate digestion and absorption. This reduces postprandial blood glucose peaks, thereby mitigating excessive stimulation of pancreatic β-cells. Additionally, LLE suppresses lipase activity to decrease dietary fat absorption, alleviating lipotoxicity interference with insulin signaling pathways, a mechanism similar to that of the OL group but with more pronounced effects. LLE significantly reduced levels of pro-inflammatory factors such as IL-1β, IL-6, and TNF-α, which can interfere with insulin signaling by inducing serine phosphorylation of insulin receptor substrate (IRS) through activation of JNK and IKK kinases. This indirectly promotes normal insulin signaling. HOMA-IR results were consistent with insulin sensitivity. Compared to the orlistat group, the LLE-H group exhibited significantly lower HOMA-IR (3.2 vs. 4.3), indicating its improved efficacy in reversing insulin resistance beyond that of a simple lipase inhibitor. This aligns with its multi-targeted mechanism of action. The reduction in HOMA-IR suggests decreased risk of type 2 diabetes. Combined with comprehensive improvements in lipid profiles, liver function, inflammatory markers, and oxidative stress status observed in this study, LLE shows promise as an effective natural product for preventing and treating obesity-related insulin resistance.

## 4. Conclusions

This study successfully optimized the ultrasonic-assisted extraction process of loquat leaf extract (LLE) using an ANN-GA. The optimal conditions were a surfactant concentration of 0.6%, ultrasonic power of 328 W, and extraction temperature of 49 °C. These conditions significantly reduced energy consumption and auxiliary material usage while maintaining extraction efficiency. In vitro bioactivity evaluation demonstrated that LLE exhibited significant dose-dependent inhibition of both α-glucosidase and pancreatic lipase, with IC_50_ values of 3.75 μg/mL and 78.3 μg/mL, respectively. In vivo experiments demonstrated that LLE intervention dose-dependently reduced body weight gain, TC, TG, LDL-C, and FBG levels in HFD-induced obese mice while restoring HDL-C levels. Notably, the high-dose LLE group (LLE-H) achieved the following results: TC 4.70 mmol/L, TG 1.30 mmol/L, ALT 78 U/L, AST 138 U/L, MDA 5.40 nmol/mL, SOD 190 U/mL, and HOMA-IR 3.2. In summary, ANN-GA-optimized LLE demonstrates promising lipid-lowering, hypoglycemic, hepatoprotective, and antioxidant activities in the in vitro enzymatic assays and the HFD-induced obese mouse model employed in this study. Although these findings highlight its intervention potential, they remain strictly preclinical in nature and do not yet support conclusions about translational efficacy in humans. Further mechanistic investigations (e.g., target identification and pathway elucidation), long-term toxicity assessments, and well-controlled clinical trials are indispensable before any application of LLE in obesity management can be realistically considered. Nonetheless, this study provides a robust experimental basis and an efficient process optimization strategy for the future development of loquat leaf-derived bioactive products.

## Figures and Tables

**Table 1:** Central composite design (CCD) and experimental results.

Samples	COS (%)	UP (W)	ET (°C)	Total Flavonoids (mg/g)	Total Phenols (mg/g)	Ursolic Acid (%)	OS
1	1.2	325	55	24.6	48.2	2.95	0.619
2	0.6	325	55	20.1	42.5	2.42	0
3	0.8	250	65	22.3	44.8	2.68	0.33
4	1.2	325	55	24.8	48	2.93	0.665
5	1.6	250	45	23.5	47.1	3.05	0.703
6	1.2	325	55	24.4	47.9	2.91	0.634
7	1.2	325	40	22.9	45.3	2.75	0.42
8	0.8	400	65	23.8	46.5	2.87	0.576
9	1.6	400	45	26.2	50.1	3.22	0.95
10	1.2	325	55	24.7	48.4	2.97	0.694
11	1.2	200	55	21.5	43.6	2.58	0.192
12	0.8	250	45	21.1	43	2.5	0.108
13	1.2	325	55	24.5	47.8	2.94	0.656
14	1.8	325	55	25	48.8	3.08	0.805
15	1.6	250	65	24.1	47.5	3.1	0.787
16	1.2	325	70	23.5	46	2.85	0.524
17	0.8	400	45	22.5	45.1	2.8	0.41
18	1.2	325	55	24.9	48.6	2.99	0.696
19	1.2	450	55	25.5	49.5	3.05	0.777
20	1.6	400	65	26.8	51.2	3.25	0.985

## Data Availability

The original contributions presented in this study are included in the article. Further inquiries can be directed to the corresponding author.
